# Supplementary material for: Numerical Study and Structural Optimization of Water-Wall Temperature-Measurement Device for Ultra-Supercritical Boiler
Source: Sensors (Basel). 2024 Sep 18;24(18):6038. doi: 10.3390/s24186038 (PMC11435903; doi:10.3390/s24186038)
Supplement: Supplementary file 1 [file sensors-24-06038-s001.zip › sensors-3185844-supplementary.pdf]

Numerical Study and Structural Optimization of Water-Wall Temperature-Measurement Device for Ultra-Supercritical Boiler

Zifu Shi, Pei Li *, Yonggang Zhou and Song Ni

Zhejiang University, Hangzhou, CN 310027

* princeleego@zju.edu.cn

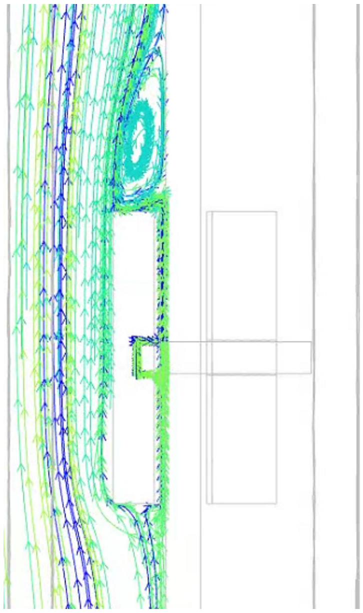

Figure S1. Flue gas flow line near the heat-collecting block within the furnace.

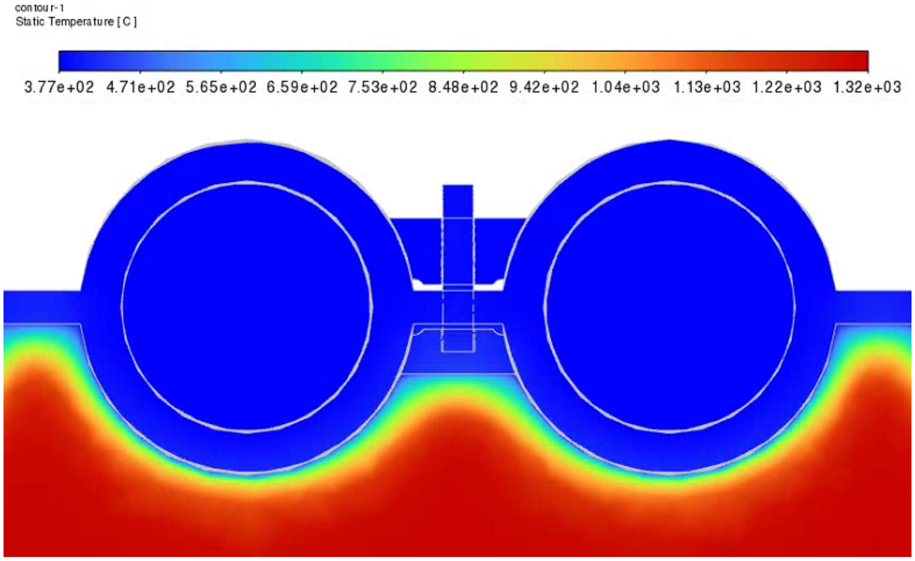

Figure S2. Temperature gradient of the flue gas near the water wall.

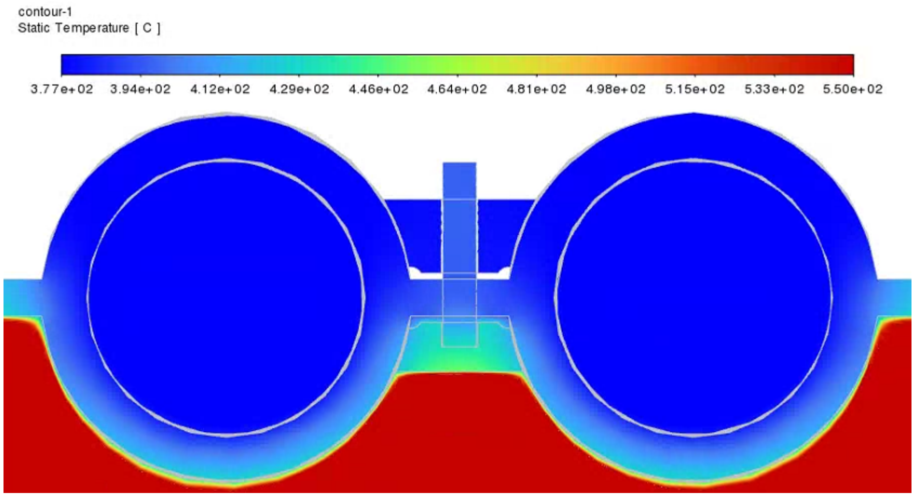

Figure S3. Temperature gradient of the heat-collecting block and water wall
